# Stable Expression of Human Muscle-Specific Kinase in HEp-2 M4 Cells for Automatic Immunofluorescence Diagnostics of Myasthenia Gravis

**DOI:** 10.1371/journal.pone.0083924

**Published:** 2014-01-09

**Authors:** Sandra George, Silvia Paulick, Ilka Knütter, Nadja Röber, Rico Hiemann, Dirk Roggenbuck, Karsten Conrad, Jan-Heiner Küpper

**Affiliations:** 1 Faculty of Science, Brandenburg University of Technology Cottbus-Senftenberg, Senftenberg, Germany; 2 GA Generic Assays GmbH, Dahlewitz/Berlin, Germany; 3 Institute of Immunology, Technical University Dresden, Dresden, Germany; Istanbul University, Turkey

## Abstract

Muscle-specific kinase (MuSK) belongs to the nicotinic acetylcholine receptor complex which is targeted by pathogenic autoantibodies causing Myasthenia gravis. While up to 95% of patients with generalized Myasthenia gravis were shown to be positive for acetylcholine receptor-specific autoantibodies, up to 70% of the remaining patients develop autoantibodies against MuSK. Discrimination of the autoantibody specificity is important for therapy of Myasthenia gravis. Recently, the new automatic fluorescence assessment platform AKLIDES has been developed for immunofluorescence-based diagnostics of autoimmune diseases. In order to establish an AKLIDES procedure for the detection of MuSK-specific autoantibodies (anti-MuSK), we developed a recombinant HEp-2 cell clone expressing the human MuSK cDNA. Here we show at the mRNA and protein level that the cell clone HEp-2 M4 stably expresses human MuSK. We provide evidence for a localization of MuSK at the cell membrane. Using cell clone HEp-2 M4 on the AKLIDES system, we investigated 34 patient sera that were previously tested anti-MuSK positive by radioimmunoassay as positive controls. As negative controls, we tested 29 acetylcholine receptor-positive but MuSK-negative patient sera, 30 amytrophic lateral sclerosis (ALS) patient sera and 45 blood donors. HEp-2 M4 cells revealed a high specificity for the detection of MuSK autoantibodies from 25 patient sera assessed by a specific pattern on HEp-2 M4 cells. By using appropriate cell culture additives, the fraction of cells stained positive with anti-MuSK containing sera can be increased from 2–16% to 10–48%, depending on the serum. In conclusion, we provide data showing that the novel recombinant cell line HEp-2 M4 can be used to screen for anti-MuSK with the automatic AKLIDES system.

## Introduction

With a prevalence of about 100–200 cases per million individuals, Myasthenia gravis (MG) is a relatively rare autoimmune disease with a trend towards increasing cases [1]. The hallmark of MG is weakness and fatigability of the skeletal muscle due to a failure of the signaling pathway at the neuromuscular junction. In about 70–95% of patients with generalized MG, failure in the neuromuscular transmission at neuromuscular junction is caused by autoantibodies targeting the acetylcholine receptor (AChR) [2]–[7]. The muscle-specific receptor tyrosine kinase (MuSK) is functionally linked to AChR triggering its membrane clustering upon association of low density lipoprotein receptor-related protein 4 with MuSK. MuSK signaling involves casein kinase 2, downstream of tyrosin kinase 7 and rapsyn. Formation of autoantibodies against AChR (anti-AChR) can be detected in up to 95% of patients with generalized MG symptoms while about 70% of the remaining patients are diagnosed positive for MuSK-specific autoantibodies (anti-MuSK) [8]–[12]. The remaining MG patients show neither binding of autoantibodies to AChR nor to MuSK. They are declared as double-seronegative MG [13].

In addition to physical and electrophysiological examinations on muscular fatigability, MG can be diagnosed by serological tests such as radioimmunoassays (RIA) detecting anti-AChR and anti-MuSK. RIAs were considered to be the gold standard. However, there is evidence that RIAs, which are based on purified autoantigens, might have reduced sensitivity for those pathognomonic autoantibodies that recognize their corresponding antigenic targets in their natural membrane environment [14]. By using immunofluorescence assays with transiently transfected cells expressing these autoantigenic targets in their natural environment, further anti-AChR and anti-MuSK positive patient sera could be identified in patients who originally were tested seronegative by RIA [15], [16]. To substitute the radioactivity-based standard assay and to enable improved analyses of autoantibody binding to receptors in their physiological conformation, we set out to develop HEp-2 cell cultures expressing proteins of the AChR complex. We choose HEp-2 cells as they represent the standard cell line for automated screening and differentiation of non-organ specific autoantibodies [17]. Here, we focus on the generation and characterization of a novel HEp-2 M4 line which constitutively overexpresses human MuSK.

Obviously, these cells expose MuSK at the cytoplasm membrane as shown by indirect immunofluorescence with non-fixed cells. In a first attempt, 34 MG patient sera which had been pretested by RIA to be MuSK-positive were investigated with the new cell-based immunofluorescence assay on the AKLIDES system. While control sera were negative, 25 MuSK autoantibody patient sera showed reactivity with HEp-2 M4. In summary, the new cell line HEp-2 M4 could be a useful biological tool for the establishment of an automatic immunofluorescence test for anti-MuSK diagnostics avoiding the use of radioactivity.

## Materials and Methods

### Cell culture and growth curve analysis

HEp-2 (human epidermoid laryngeal carcinoma) cells (ATCC: CCL-23) were routinely cultivated in growth medium, *i.e.* Dulbecco's MEM medium (Biochrom AG, Berlin, Germany) supplemented with 10% fetal bovine serum (GE Healthcare, Austria), 2 mM L-Alanyl-L-Glutamine, 1× MEM non-essential amino acids, and 1 mM MEM sodium pyruvate (each Biochrom AG) at 37°C and 5% CO_2_ in a humidified incubator. Medium was changed every two to three days and sub-cultivation was performed at 80–90% confluence.

For routine passaging, the HEp-2 M4 clone was cultivated in growth medium. In order to further stimulate MuSK expression, cells were cultivated in growth medium with epigenetics supplement mix (ESM, available at Medipan GmbH, Dahlewitz, Germany). For expression analyses, cells were seeded in appropriate cell numbers such as 1.3×10^4^/cm^2^ for HEp-2 cells, 2.0×10^4^/cm^2^ for HEp-2 M4 cells and 3.2×10^4^/cm^2^ for HEp-2 or HEp-2 M4 cells grown in medium containing the supplement mix. Cells were grown for 48 h under standard cultivation conditions.

For growth curve analyses, cells were trypsinized and 2.1×10^3^ cells per cm^2^ were plated in triplicate into 6-well plates (TPP, Trasadingen, Switzerland). Cells were further cultured under standard conditions. To overcome the lag phase, cells were counted every 24 h by a hemacytometer, starting after 72 h. The doubling time was calculated from plot of cell numbers against time (Origin 7.5G program, Massachusetts, USA).

### Generation of HEp-2 cells with stable expression of MuSK

A retrovirus expression vector was designed which contains the human cytomegalovirus promoter controlling the open reading frame of MuSK. MuSK cDNA was synthesized without stop codon according to NCBI deposition #AF006464.1. Upon expression, MuSK is carboxy-terminally fused to the V5 epitope tag (GKPIPNPLLGLDST) provided by the vector to allow autoantibody-independent detection of MuSK expression.

Recombinant retroviruses were produced according to standard methods and used to infect HEp-2 cells. Stable transfectants were screened for MuSK expression by using an anti-MuSK positive patient serum in indirect immunofluorescence.

### RT-PCR

After a 48 h cultivation period, cells were washed with phosphate-buffered saline (PBS) and lysed with TriFast (PEQLAB Biotechnology GmbH, Erlangen, Germany). Total RNA was prepared according to the manufacturer's protocol.

Total RNA was pre-treated with *Dnase*I (ThermoScientific, Waltham, USA). Reverse transcriptase reaction was performed according to manufacturer's protocol using RevertAid First Strand cDNA Synthesis Kit and oligo(dT) primer (ThermoScientific). The cDNA was used for PCR analysis with forward primer 5′- CCCAGTTTCACCAGTATTCAC-3′ and reverse primer 5′- GTAGAATCGAGACCGAGGAG-3′. Oligonucleotide primers were synthesized by BioTez (Berlin, Germany). PCR reactions were done using probe qPCR master mix (ThermoScientific). PCR was performed as the following: initial step 10 min 95°C; 40 cycles with 20 sec 94°C, 25 sec 59.5°C, 1 min 72°C with Mastercycler epgradient (Eppendorf AG, Wesseling-Berzdorf, Germany). Amplicons were analyzed by 3% agarose gel electrophoresis and compared to 50 bp marker (Roth, Karlsruhe, Germany).

For expression analysis, quantitative real-time PCR was performed with iQ™5 Thermo Cycler (Bio-Rad Hercules, USA). Data were normalized to reference gene GAPDH [18] by Rest2009 software (Qiagen, Hilden, Germany).

### Western Blot analysis

Cells were trypsinized and spinned down; pellets were dissolved in Laemmli extraction buffer at 95°C. Protein extracts of 2×10^5^ cells per lane were subjected to a 10% Tris/HCl sodium dodecyl sulfate-polyacrylamide gel electrophoresis [19] and transferred to a Roti® PVDF membrane (Roth). After blocking, the membrane was incubated with anti-V5 monoclonal antibody (Life Technologies, Darmstadt, Germany) diluted 1∶2,000 at 4°C for 16 h. Washings were performed with PBS supplemented with 0.3% Tween-20 at room temperature (RT). Peroxidase–conjugated goat anti-mouse IgG (Sigma-Aldrich, diluted 1∶500) was incubated for 1 h at RT followed by 5× washing. The membrane was incubated with ECL™ Prime Western Blotting Reagents (GE Healthcare, Freiburg, Germany) for 5 min in the dark, and chemiluminescence was analyzed after 5 sec exposure time with the LumiImager F1 system (Roche Diagnostics) and LumiAnalyst program. Protein sizes were compared to PageRuler Plus Prestained Protein Ladder (Thermo Scientific).

### Indirect Immunofluorescence

Cells were trypsinized, and 2.5×10^3^ HEp-2 cells, 4×10^3^ HEp-2 M4 (cultivated in growth medium), or 6.3×10^3^ HEp-2 M4 cells (cultivated in growth medium plus supplements), respectively, were seeded onto diagnostic glass slides (ThermoScientific). After cultivation for 48 h, indirect immunofluorescence was performed as described [16]. Briefly, living cells were incubated with MG patient sera (diluted 1∶20 with 1% bovine serum albumin [BSA] in PBS), or anti-MuSK antibody (Abcam, Cambridge, UK, diluted 1∶200 with 1% BSA in PBS) for 1 h at RT. Cells were washed in PBS and immediately fixed with 3% formalin for 15 min at RT, washed with PBS and incubated with methanol (−20°C) for 2 min. To detect MuSK, fixed cells were labeled with biotin-conjugated goat anti-human IgG (Dianova, Hamburg, Germany) diluted 1∶1,000 with 1% BSA in PBS followed by incubation with streptavidin Alexa Fluor488 (final concentration 0.5 µg/ml, Dianova) including 2 µg/ml DAPI (Roth). To detect the fused V5 epitope tag, fixed cells were incubated with anti-V5 monoclonal antibody (Life Technologies) diluted 1∶200 with 1% BSA in PBS followed by incubation with Cy3-conjugated goat anti-mouse IgG (Dianova) diluted 1∶400 with 1% BSA in PBS. For double immunofluorescence staining analyses, after patient serum incubation and cell fixation, anti-V5 primary antibody was combined with biotin-conjugated goat anti-human IgG. Thereafter, cells were incubated with Cy3-conjugated goat anti-mouse IgG combined with streptavidin Alexa Fluor488. After staining, slides were washed in PBS, mounted in fluorescence mounting medium (DakoCytomation, Hamburg, Germany) and stored at 4°C in the dark.

For routine fluorescence microscopy assessments of patient sera reactivity with HEp-2 M4 cells, the automatic fluorescence interpretation platform AKLIDES (Medipan GmbH, Berlin-Dahlewitz, Germany) was used [20], [21] with modus TRANSFECT. At least two independent experiments per condition were performed. AKLIDES analysis resulted in four pictures per approach. Positive cells were related to total cell number.

For double immunofluorescence analyses, IX81 fluorescence microscope (Olympus, Tokyo, Japan) combined with the xenon burner MT20 (Olympus) was used. Pictures were taken with F-View II camera (Olympus) and documented with Olympus cellR-imaging software.

Routine cell culture pictures were taken with the phase contrast microscope CKX41 (Olympus) and documented with Olympus cellF-imaging software.

### MG patient sera

Patient sera were kindly provided by Professor R.-L. Humbel (LLAM, Luxembourgeois d'Immuno-Pathologie) and by K. Conrad (Institute of Immunology at Technical University of Dresden). All used sera in this study were pretested by ^125^I-MuSK and immunoprecipitation assay (DLD Diagnostika GmbH, Hamburg, Germany) and analyzed with the 1470 Wizard gamma- scintillation counter (serial number 4701494, Perkin Elmer, Massachusetts, USA). The sera in this study were used according to ethics committee vote (EK 226112006) of the Technical University Dresden (Ethics Commission of the Medical Faculty Carl Gustav Carus of the Technical University Dresden). An approval of the donors was not necessary because fully anonymized probes used as quality controls in routine diagnostics were selected for this study only.

### FACS analysis

Cells were cultivated for 48 h, trypsinized and counted. Cells (1×10^6^) were centrifuged at 300 g for 5 min at room temperature. The supernatant was discarded and the pellet was resuspended in 1 mL PBS. A fixation buffer of 70% (v/v) 4°C ethanol in PBS was added to the cell suspension and cells were stored at 4°C for at least 1 h. Prior to the measurement, the ethanol-fixed cells were centrifuged at 300 g for 5 min. The pellet was dissolved in 1 µg/mL DAPI in PBS followed by incubation for 10 min at 4°C in the dark [22]. The samples were transferred to flow cytometer (BD FACSCantonTM II, BD Biosciences, San Jose, USA), and cell fluorescence was measured (solid-state diode ex 405 nm, em 550/50 nm). The percentages of cells in specific cell cycle phases (G0/G1, S or G2/M) were determined from histograms by region integration using FlowJo 7.6.5 (Oregon, USA) data analysis application.

### Statistical analysis

Mean standard derivation, t-test and Mann-Whitney U test were calculated with the Origin 8.6 program. A p value of <0.05 was considered statistically significant. Immunofluorescence signals obtained by the AKLIDES system were scored semi quantitatively as the following: −, negative; +/−, weak fluorescence signal; ++, medium signal; +++, strong signal.

### Results

In order to improve MG serological diagnostics and to substitute for the standard RIA, we attempted to establish an automatic indirect immunofluorescence assay based on stable transfectants of the standard cell line HEp-2 genetically engineered to express proteins of the AChR complex. In this study we focus on the diagnostics of anti-MuSK, targeting a key player for AChR clustering.

### Generation of cell clone HEp-2 M4 with stable expression of human MuSK

HEp-2 cells were infected with a recombinant retrovirus enabling constitutive expression of the human MuSK cDNA. Four weeks after infection, cell clones resistant to the selection antibiotic blasticidin could be isolated. Clones were subjected to single cell cloning and screened for MuSK expression by indirect immunofluorescence using an anti-MuSK positive MG patient serum. Out of 45 isolated antibiotic-resistant cell clones only five cell clones clearly showed anti-MuSK binding. Since retroviruses usually integrate *via* their long terminal repeats as complete proviruses into the host cell genome, MuSK expression cassettes very likely were preserved intact in the remaining 40 clones. Thus, lack of MuSK expression in those clones could be due to gene silencing processes or unfavorable genomic integration sites.

**Figure 1:**
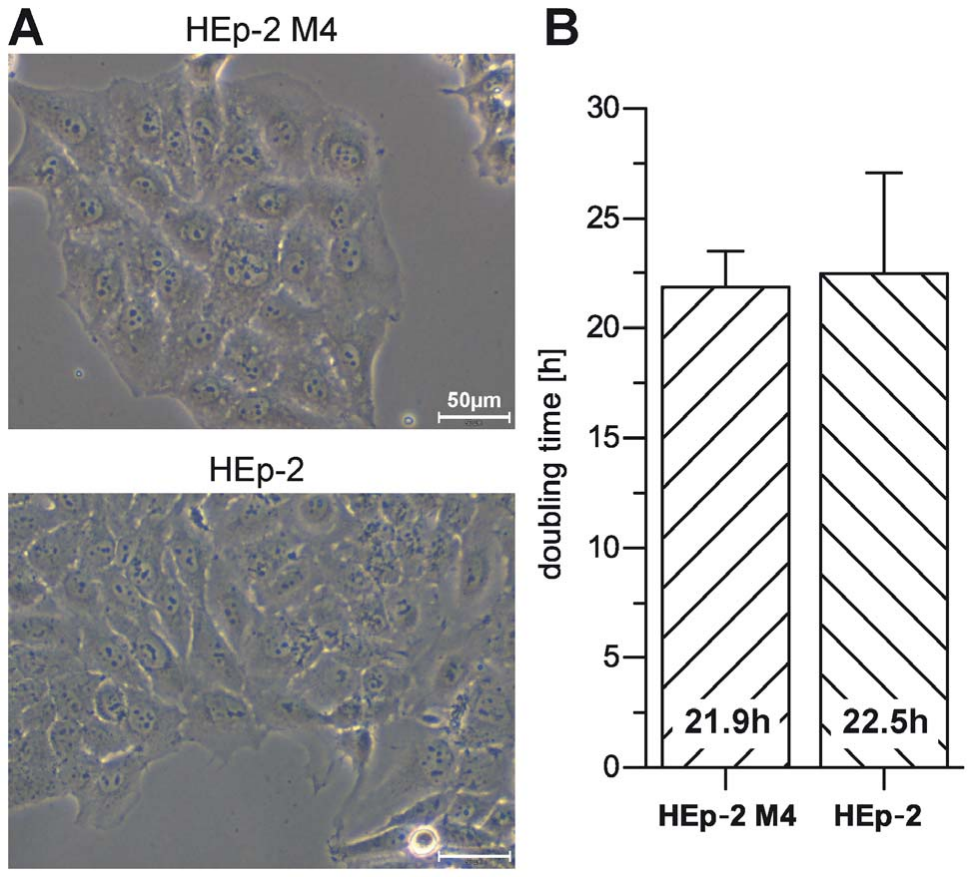
HEp-2 M4 cells keep morphology and proliferation characteristics of parental HEp-2 cells. A. Phase contrast images of exponentially growing transfected HEp-2 M4 and non-transfected HEp-2 parental cells. Images were obtained with microscope CKX41 and 10× objective (scale bar 50 µm). B. Cell doubling times of exponentially growing cultures of both cell lines were determined as described in materials and methods.

Of those clones that were found positive for MuSK expression, cell clone HEp-2 M4 was chosen for further analyses due to its strong MuSK expression. As shown in Figure 1A, cell clone HEp-2 M4 retained the typical cell morphology of the HEp-2 parental cell line. Both cell lines were composed of typical spherical cells (Figure 1A). Interestingly, also cell doubling times were almost identical between HEp-2 M4 and parental HEp-2 cells (Figure 1B). These results indicated that HEp-2 M4 cells can be handled as convenient as HEp-2 cells.

**Figure 2:**
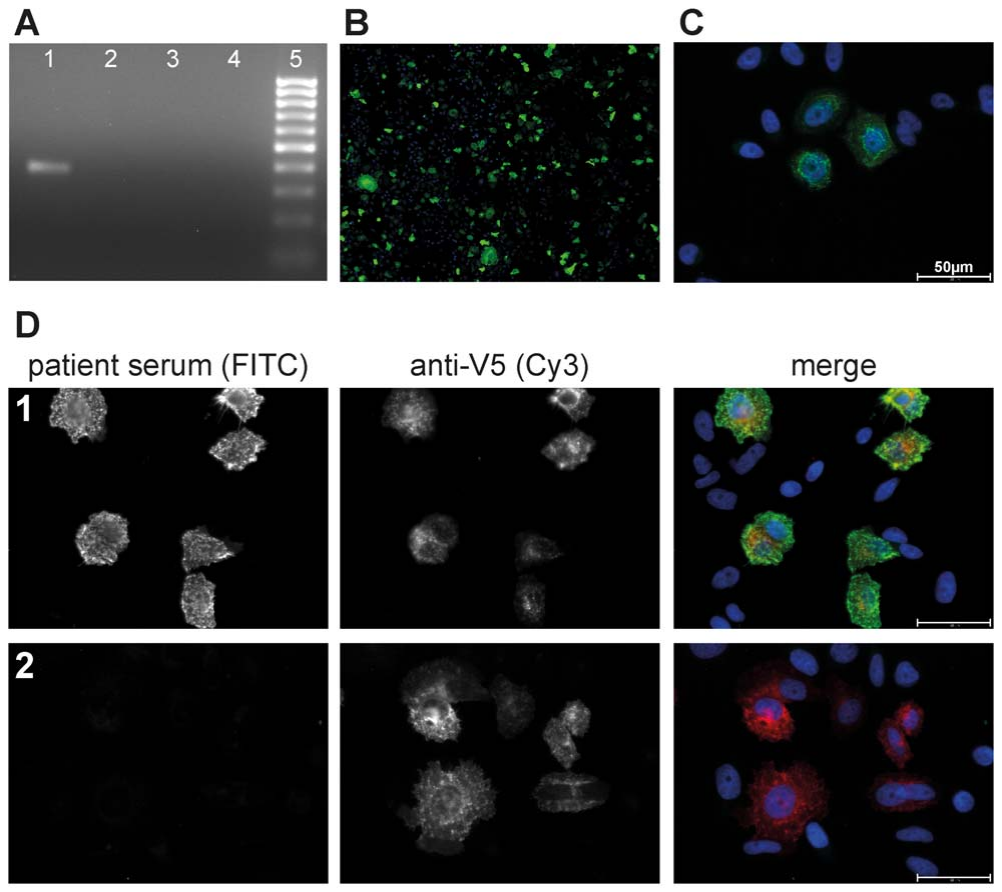
Detection of MuSK expression in HEp-2 M4 cells. A. Exponentially growing transfected clone HEp-2 M4 and non-transfected parental HEp-2 cells were used for RNA preparation and RT-PCR analysis of heterologously expressed MuSK, with (+) or without (−) reverse transcriptase (RT), as described in materials and methods. 1, HEp-2 M4+RT; 2, HEp-2 M4−RT; 3, HEp-2+RT; 4, HEp-2−RT. Amplicons were analyzed by agarose electrophoresis with 50 bp DNA size marker as control (5). B. HEp-2 M4 cells growing on glass slides were fixed and processed for indirect immunofluorescence with anti-V5 antibody to detect expression of the MuSK-V5 fusion protein as described in the methods section. Photo was taken by AKLIDES system (10× objective, TRANSFECT mode). C. Living HEp-2 M4 cells grown on glass slides were incubated with commercial anti-MuSK primary antibody followed by fixation and secondary antibody staining as described in the methods section. The image was taken by an Olympus fluorescence microscope IX81 (40× objective). MuSK-specific signals appear as characteristic speckled pattern. D. Living HEp-2 M4 cells grown on glass slides were incubated with anti-MuSK autoantibody positive patient sera (1) and further processed for double immunofluorescence staining as described in materials and methods. A second serum pretested by RIA to be negative for anti-MuSK autoantibody (2) was used as negative control. Detection of the V5 epitope by V5-specific primary antibody and Cy3 labeled secondary antibodies, and simultaneous detection of MuSK with patient autoantibodies and Alexa Fluor 488 conjugated secondary antibodies is illustrated as indicated with V5 (Cy3) and patient serum (FITC channel used), respectively. Double immunofluorescence staining images were obtained by merging both images as indicated (merge). The double immunofluorescence staining analysis shows strong evidence for specific binding of MuSK autoantibodies to only those HEp-2 M4 cells expressing the MuSK-V5 fusion protein. Images were taken by the Olympus fluorescence microscope IX81 (40× objective). Scale bar 50 µm.

Within the used retrovirus vector construct, MuSK cDNA is fused in frame to the coding sequence of the V5 epitope. In order to demonstrate heterologous human MuSK transcription, we used RT-PCR with forward and reverse primer binding sites in the MuSK cDNA and V5 epitope sequences, respectively. Figure 2A shows formation of a specific RT-PCR amplicon for HEp-2 M4 but not for HEp-2 control cells.

MuSK expression in HEp-2 M4 cells was further analyzed by V5 epitope-specific indirect immunofluorescence. Around 20% of HEp-2 M4 cells were detected positive for the V5 epitope (Figure 2B) with different signal intensities in those positive cells. Despite single cell cloning, the majority of cells does not seem to express the V5-tagged MuSK. This could be due to posttranslational mechanisms reducing the stability of the MuSK protein or due to downregulation of MuSK expression at the promoter level by epigenetic processes.

### Autoantibody binding to non-fixed cells

In order to increase the sensitivity of the immunofluorescence test, we employed a protocol where MG patient sera were incubated with living instead of fixed cells. Thus, the main autoantigenic epitopes of MuSK residing within the extracellular part of this receptor protein should be better recognized by autoantibodies if preserved in their native conformation. A similar protocol was used by Leite and colleagues [16] to detect autoantibodies against the AChR complex including MuSK. A further advantage of using living cells is that patient sera antibodies should not penetrate through the plasma membrane avoiding their unspecific binding to intracellular structures. Non-accessibility of internal cell structures has been tested by incubation of living HEp-2 M4 cells with strongly positive anti-nuclear autoantibodies sera diluted 1∶20 (data not shown). Proper MuSK presentation at the plasma membrane of living cells was tested by using a commercially available anti-MuSK antibody. Indeed, by using this antibody in indirect immunofluorescence, MuSK expression typically appeared as punctuated pattern which could be localized at the plasma membrane (Figure 2C). However, with the used microscopic equipment we cannot directly prove plasma membrane localization.

In order to further show that autoantibodies of patient sera do specifically bind to MuSK-expressing cells, we performed a double immunofluorescence staining based on the availability of the fused V5 tag (Figure 2D). The respective image clearly shows that all cells positive for autoantibody binding were also positive for binding of the anti-V5 antibody. Altogether, HEp-2 M4 cells express sufficient amounts of MuSK which can be readily detected by indirect immunofluorescence if MG patient sera are exposed to living cells.

### Optimization of MuSK expression in HEp-2 M4 cells

First experiments showed MuSK-specific autoantibody binding in less than 20% of HEp-2 M4 cells. To obtain a higher fraction of MuSK-expressing cells, we tested a commercially available epigenetics supplement mix (ESM). It is a well-known phenomenon that expression of some genes can be silenced by methylation of CpG islands within promoters which in turn *via* methyl CpG-binding domain proteins leads to the attraction of histone deacetylases and chromatin remodeling [23], [24]. Accordingly, treatment of cells with substances that interfere with epigenetic silencing processes can lead to the rescue of gene expression [25].

**Figure 3:**
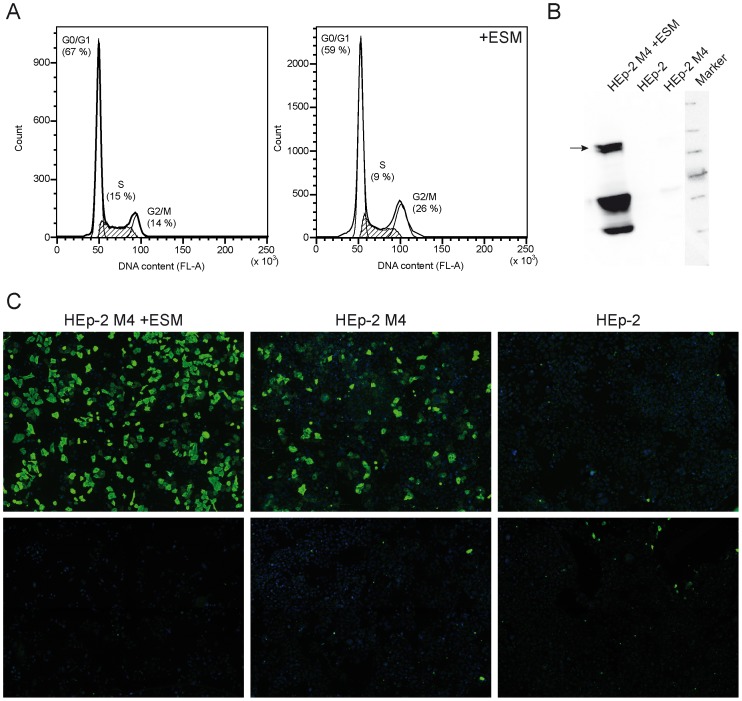
ESM increases MuSK expression in HEp-2 M4 cells. A. Exponentially growing HEp-2 M4 cells were treated (right chart) or not treated (left chart) with the epigenetic supplement mixture (ESM) as indicated. Cell cycle phases were analyzed by FACS. Although there is an increase of cells in G2/M phase with ESM, this treatment apparently does not induce cell cycle arrest. B. Cell extracts from exponentially growing transfected HEp-2 M4 or untransfected parental HEp-2 cells were processed for Western Blotting. Cells had been treated or not with the epigenetic supplement mixture (ESM) as indicated. Detection of the MuSK-V5 fusion protein was performed as described in the materials section. Expected band of expressed MuSK is indicated by arrow, other bands are probably degradation products. C. Clone HEp-2 M4 or parental HEp-2 cells were treated or not with ESM as indicated, and MuSK expression was analyzed by immunofluorescence using MuSK autoantibody-positive patient serum (1). A serum pretested by radioimmunoassay to be negative for anti-MuSK autoantibodies was used as control (2). Immunofluorescence images were evaluated by AKLIDES system (10× objective, TRANSFECT modus).

Analysis at the mRNA level by qRT-PCR revealed that MuSK expression indeed could be upregulated about tenfold (p = 0.003) when cells were treated with the ESM. Since expression of tumor suppressor proteins can be silenced during tumor formation, their rescue by substances reducing DNA methylation and interfering with histone deacetylation potentially can lead to cessation of cell proliferation in established tumor cell lines [26]. We therefore analyzed possible side effects of the ESM on cell cycle by using flow cytometry. Indeed we found that supplement treatment of clone HEp-2 M4 reduced the fraction of S-phase cells and increased that of cells at G2/M transition (Figure 3A), but there was no cell cycle arrest. In line with increased MuSK mRNA levels after supplement treatment, we also detected a dramatic increase of protein levels thereof (Figure 3B). The immunoblotting data revealed some evidence of proteolytic MuSK degradation which might result in reduction of available MuSK epitopes at the plasma membrane. Nevertheless, indirect immunofluorescence analysis with an anti-MuSK specific MG patient serum showed a strong increase of positive signals in HEp-2 M4 cells treated with the ESM (Figure 3C).

### Specific detection of anti-MuSK autoantibodies with HEp-2 M4 cells

To analyze sensitivity and specificity of anti-MuSK detection with HEp-2 M4 cells, we tested 63 sera from MG patients. Among these MG sera, 34 sera were found by RIA to contain MuSK-specific autoantibodies (anti-MuSK group) and 29 sera were found by RIA to contain AChR-specific autoantibodies (anti-AChR group) but no anti-MuSK reactivity. As controls we used 30 sera from ALS patients and 45 sera from healthy blood donors. As shown in Table 1, 25 out of the 34 anti-MuSK sera were also positive by the cell based assay. Below a certain RIA value (790 pmol/L) the cell based assay did not yield positive result, except one serum (see below).

**Table 1:** MuSK autoantibodies positive patient sera listed according to RIA values.

Patient serum	RIA [pmol/L]	HEp-2 M4+ESM			HEp-2 M4−ESM			p values	
		X¯	±SD	FI	X¯	±SD	FI	p_1_	p_2_
1	343400	0.288	0.048	+	0.074	0.011	+	2.8E-5	2.0E-4
2	179600	0.371	0.076	+++	0.162	0.111	++	4.0E-4	2.1E-4
3	2300	0.425	0.129	+++	0.116	0.025	++	2.8E-5	2.7E-4
4	2205	0.280	0.114	+++	0.066	0.019	++	1.3E-4	4.3E-4
5	2189	0.288	0.094	+++	0.075	0.013	++	1.2E-4	4.5E-4
6	2176	0.290	0.135	+++	0.063	0.008	++	1.2E-4	2.8E-4
7	2107	0.368	0.063	+++	0.084	0.004	+	1.2E-4	5.0E-4
8	1984	0.351	0.107	++	0.063	0.016	+	2.8E-5	1.6E-4
9	1920	0.240	0.116	++	0.047	0.024	+	1.2E-4	2.0E-4
10	1890	0.413	0.177	+	0.124	0.026	+	4.7E-4	3.7E-3
11	1861	0.467	0.019	++	0.084	0.003	+	1.2E-4	3.9E-4
12	1844	0.333	0.083	++	0.066	0.010	++	1.2E-4	4.5E-4
13	1788	0.302	0.089	+++	0.052	0.002	++	1.2E-4	2.8E-4
14	1785	0.485	0.031	+++	0.089	0.002	+	1.2E-4	3.4E-4
15	1766	0.259	0.055	++	0.062	0.001	++	1.2E-4	3.4E-4
16	1752	0.277	0.059	+++	0.060	0.011	++	1.2E-4	3.9E-4
17	1750	0.428	0.038	+++	0.082	0.017	++	1.2E-4	4.3E-4
18	1729	0.333	0.126	++	0.064	0.005	+	1.2E-4	6.5E-3
19	1641	0.434	0.069	+++	0.078	0.031	++	1.2E-4	3.9E-4
20	1592	0.469	0.064	+++	0.088	0.028	+	1.2E-4	3.4E-4
21	1592	0.333	0.039	+	0.079	0.008	+/−	2.8E-5	1.2E-4
22	1491	0.268	0.070	+++	0.063	0.022	+	2.2E-3	0.0997
23	837	0.105	0.063	+	0.019	0.001	+/−	1.1E-3	4.2E-3
24	791	0.255	0.120	+/−	0.022	0.013	+/−	2.2E-4	4.1E-3
25	580	n.v.		−	n.v.		−	n.a.	n.a.
26	194	n.v.		−	n.v.		−	n.a.	n.a.
27	170	n.v.		−	n.v.		−	n.a.	n.a.
28	112	n.v.		−	n.v.		−	n.a.	n.a.
29	110	n.v.		−	n.v.		−	n.a.	n.a.
30	90	n.v.		−	n.v.		−	n.a.	n.a.
31	87	0.348	0.115	+	0.054	0.010	+/−	2.8E-5	2.1E-3
32	80	n.v.		−	n.v.		−	n.a.	n.a.
33	79	n.v.		−	n.v.		−	n.a.	n.a.
34	70	n.v.		−	n.v.		−	n.a.	n.a.

**Figure 4:**
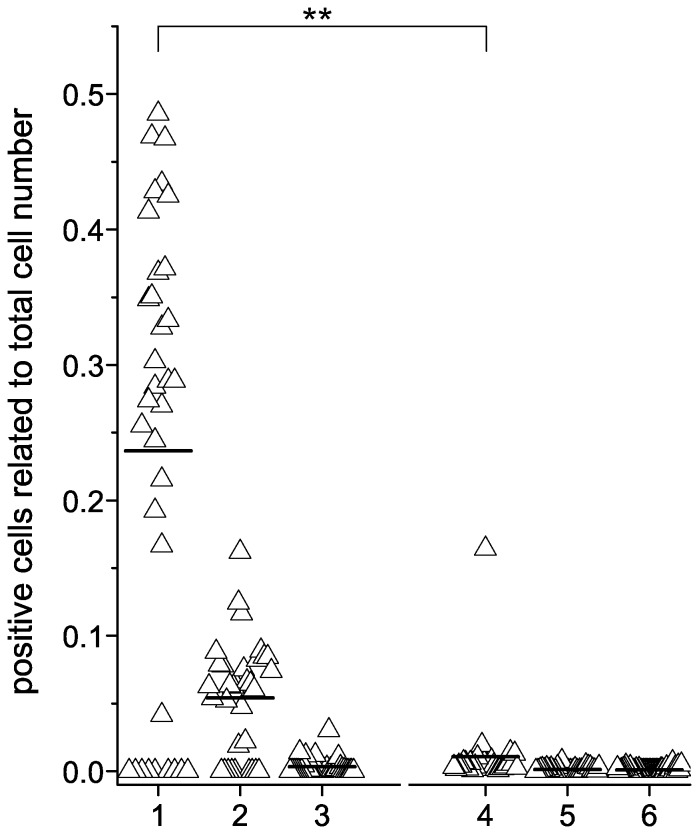
Anti-MuSK detection on HEp-2 M4 cells. HEp-2 M4 cells were seeded onto glass slides, and living cells were cultivated in the presence or absence of ESM. Cells were incubated with MG patient sera pretested by RIA to contain anti-MuSK autoantibodies or with control sera. Cells were further processed for indirect immunofluorescence testing by using the protocol described in the materials section along with AKLIDES system (10× objective, TRANSFECT modus). Each experiment was repeated two or three times, respectively. Four photographs were taken automatically by AKLIDES from each well and evaluated manually as described in in the materials section. Anti-MuSK autoantibody binding of HEp-2 M4 treated with ESM (1) was compared to HEp-2 M4 (2) and HEp-2 cells (3), both without ESM treatment. ESM-treated HEp-2 M4 were incubated with MG patient sera positive for anti-AChR (4), sera from ALS patients (5) and sera from healthy blood donors (6). The control groups were also tested on HEp-2 M4−ESM as well as on HEp-2 cells, but only the results for HEp-2 M4+ESM are shown. The median is indicated as line. ESM-treated HEp-2 M4 cells incubated with anti-MuSK antibodies showed significantly more positive cells compared to AChR control group (p value = 2.7E-5).

The table provides data from Figure 4. MuSK autoantibody positive patient sera are shown for HEp-2 M4+ESM and HEp-2 M4−ESM. The sera are sorted to descending RIA values (RIA cut off: >50 pmol/L positive, >500 pmol/L strong positive). The value X¯ describes the mean value of positive cells related to total cell number which is combined with standard deviation (±SD) and semi quantitatively scored fluorescence intensity (FI). The p-values are given for HEp-2 M4+ESM compared to HEp-2 M4−ESM (p_1_) and HEp-2 M4−ESM compared to HEp-2 cells (p_2_). N.v.: not valuated because FI was negative, n.a.: not available due to negative FI.

All control sera (anti-AChR positive sera, sera from ALS patients and healthy blood donors) were pretested by RIA to be negative for anti-MuSK; these control sera occasionally showed some autoantibody binding to a small fraction of HEp-2 M4 cells (data not shown). This binding pattern was mainly characterized by homogenous fluorescence signals throughout the cell but omitted the cell nuclei. We interpret this binding pattern as being unspecific since it could be clearly distinguished from punctuated anti-MuSK binding in HEp-2 M4 cells, and it was also present in HEp-2 parental cells. Figure 4 shows, that ESM-untreated HEp-2 M4 cells can be used to detect the presence of anti-MuSK in MG patient sera, but the striking effect of ESM treatment is the significant increase of the percentage of HEp-2 M4 cells which gave signals with anti-MuSK positive sera. The figure shows that the median value increased from 0.005 (Figure 4, group 2) to 0.245 (Figure 4, group 1) if cells are treated with ESM to upregulate recombinant MuSK expression. All control group sera except one were tested negative for MuSK autoantibodies. There was one serum from the AChR group that was clearly tested positive for anti-MuSK by the cell-based assay (Figure 4, group 4).

As shown in Table 1, HEp-2 M4 cells not treated with ESM showed a smaller fraction of positive stained cells (2–16% positive cells, depending on the used serum) than with those cells pretreated with ESM (10–48% positive cells). Nevertheless, already ESM-untreated HEp-2 M4 cells showed a typical anti-MuSK specific binding pattern which is not visible in the parental strain HEp-2 cells (Table 1, p2 values ≥2.1E-3, except serum 22).

Interestingly, with sera pretested above a RIA value of 790 pmol/L, RIA values don't correlate with fluorescence intensities and fractions of positive cells in the cell-based assay. Serum 1 has the highest MuSK autoantibody according to the RIA but showed only 29% of positive cells with a low fluorescence intensity compared to e.g. serum 19. Even MuSK sera with an identical RIA value such as serum 20 and 21 clearly differ in their percentages of positive cells and fluorescence intensities. Furthermore, serum 31 with a RIA value far below 790 pmol/L, here defined as threshold for all other sera of the anti-MuSK group, clearly showed positive signals in the cell based immunofluorescence assay.

## Discussion

A milestone in MG research was the discovery of autoantibodies directed against the nicotinic AChR found in the majority of MG patients. Therefore, MG was first defined by the presence of AChR-specific autoantibodies, but for those patients with clinical symptoms of MG that lack AChR autoantibodies [4] a definition of a MG subpopulation was required. Further studies showed that 5–30% of MG patients are seronegative for AChR autoantibodies [2]–[7]. In 2001, Hoch and colleagues identified MuSK as a further target for the development of autoantibodies in about 70% of MG patients with seronegativity for anti-AChR [8]. The pathogenicity of MuSK autoantibodies was confirmed by active immunization of rabbits, mice and rats with the extracellular domain of MuSK [27]–[30]. Further studies confirmed these results by injection of human total IgG purified from MuSK MG patients into test animals [31]–[34].

Testing for anti-MuSK is important for MG patients not only from a diagnostic but also from a therapeutic point of view. Anti-MuSK positive patients frequently are women below 40 years of age at the onset of disease [9], [11], [35]–[37]. Typical symptoms of these patients are severe bulbar dysfunction and respiratory insufficiency as well as atrophy of facial and tongue muscles [12], [38]–[41]. On the other hand, Caress and colleagues described in a case report, that only the ocular muscles were affected in anti-MuSK positive patients [42]. Thymic abnormalities frequently found in anti-AChR positive MG patients are absent or less pronounced in patients positive for anti-MuSK [14] diminishing the need for a thymectomy. Furthermore, MG patients negative for anti-AChR typically do not respond to anti-cholinesterase treatment; in contrast, their clinical symptoms can be aggravated with this drug [35], [36]. In standard therapy, anti-MuSK positive MG patient need a higher dosage of corticosteroids than anti-AChR positive patients, or they require additional immunosuppressive therapy [36], [43], [44]. All in all, it is of clinical importance to discriminate between patients who are seropositive for anti-MuSK or anti-AChR.

The first step towards a cell-based indirect immunofluorescence assay for the detection of anti-AChR and anti-MuSK was established by Vincent and colleagues in 2005 [45]. They used transiently transfected human embryonic kidney (HEK-293) cells for the expression of the respective proteins. We decided to express AChR components in human larynx carcinoma cell line HEp-2 being the standard cell line for automatic indirect immunofluorescence detection of autoantibodies in patients with rheumatic diseases. These cells can be easily grown on glass slides even at high confluences and, thus, provide ideal characteristics for the manufacturing of diagnostic kits. Furthermore, the HEp-2 cell derived clone M4 responded to ESM treatment with upregulation of MuSK expression without apparent toxic effects. To our knowledge, no stable cell line expressing MuSK has been established so far. In order to use the AKLIDES system for routine anti-MuSK detection, we adapted the software algorithm of the automated interpretation system [46] to membrane-associated autoantibody testing (transfect mode). So far, this allows for automatic photographs with standardized exposure times at e.g. four defined positions in each well. The next step in automation will be to develop a pattern recognition mode for membrane autoantigens and a mode for quantitation of fluorescence intensity. For the study we have used the pictures made automatically by the AKLIDES system, but the evaluation of the pictures have been performed manually as stated in the Materials and Methods section.

The AKLIDES system originally was developed for routine diagnostics of autoantibodies such as anti-nuclear and anti-neutrophilic cytoplasmic antibodies in systemic autoimmune rheumatic diseases [47]–[49]. Recently, the AKLIDES system was adapted for γH2AX foci and microbead-based immunoassays [21], [50], [51]. HEp-2 M4 immunofluorescence analysis with AKLIDES showed specific binding of anti-MuSK from MG patients. We identified two distinct immunofluorescence patterns: (I) a strong anti-MuSK specific staining revealed a speckled pattern that appeared as being concentrated around the cell nuclei, although signals should be caused by the extracellular parts of MuSK. (II) Weak unspecific signals which typically appeared more homogenously distributed with no such concentration around the cell nuclei. Importantly, those sera also showed the same signals on HEp-2 cells while obvious MuSK-specific signals were not found with those control cells.

Running anti-MuSK positive patient sera, transfected HEp-2 M4 cells but not HEp-2 showed specific binding of anti-MuSK. 24 out of 34 anti-MuSK positive sera (RIA values ≥791 pmol/L) demonstrated higher reactivity with MuSK-positive HEp-2 M4 cells after ESM treatment. Also one serum with a MuSK autoantibody concentration far below this threshold showed specific anti-MuSK binding (Table 1). Our date provide strong evidence that the sensitivity of the indirect immunofluorescence assay can be increased by ESM treatment without reducing the specificity.

The fact, that clone HEp-2 M4 expresses MuSK in only a fraction of cells (10–48% are detected in ESM-treated cells depending of the used anti-MuSK positive serum) is not a disadvantage because MuSK-positive and MuSK-negative cells can be analyzed simultaneously. Furthermore, it reflects the variability of the range of anti-MuSK binding to HEp-2 M4 cells. False positive sera can be identified when nearly all cells show fluorescence signals. Those sera would cause signals in non-transfected HEp-2 cells either which can also be used as negative control.

Notably, there does not seem to be a correlation between RIA values and AKLIDES signal intensity above a certain RIA threshold value. This raises the interesting point whether RIA or cell-based assay signals could correlate with the disease status. It has been shown that anti-MuSK concentration can drop down during immunosuppressive therapy of MG [52], [53]. But it is still an open question whether there is a correlation of anti-MuSK concentration to the disease severity of MG patients [11], [54]. Most studies have used a RIA to determine the anti-MuSK concentration. With a cell-based assay as described here both the fraction of cells stained positive for anti-MuSK binding and the fluorescence intensity can be used to investigate possible correlations with disease severities.

In particular, more sera with low anti-MuSK levels should be tested to further evaluate sensitivity and specificity of anti-MuSK binding below a RIA value of 700 pmol/L. We assume that autoantibody binding to their respective autoantigenic target on living cells in the reaction environment of an indirect immunofluorescence test mimics the in *vivo* MuSK conformation better than that in a RIA with purified protein preparations. Interestingly, we found one patient serum with anti-AChR autoantibodies which also showed anti-MuSK binding to HEp-2 M4 cells but was tested negative by RIA. This result also underlines that a cell-based assay for autoantibody detection can lead to different results than RIA which is in line with published work of Vincent and colleagues [16]. Our data appear to confirm this assumption and warrants further clinical studies.

In summary, the established clone HEp-2 M4 can represent a new cell culture model of ectopic MuSK expression which is not clustered in this artificial cell line. Here we present data on a test system based on incubation of patient sera with living cells [16] to exclude antigen modulation by fixation reagents. The development of a fixation protocol is in progress with promising and comparable results to the currently used assay. In addition, an automatic AKLIDES program is in developmental process for routine diagnostics of anti-MuSK binding.
